# Analysis of the Risk of Wear on Cemented and Uncemented Polyethylene Liners According to Different Variables in Hip Arthroplasty

**DOI:** 10.3390/ma14237243

**Published:** 2021-11-27

**Authors:** Basilio De la Torre, Loreto Barrios, Juan De la Torre-Mosquera, Julia Bujan, Miguel A. Ortega, Carlos González-Bravo

**Affiliations:** 1 Department of Surgery, Medical and Social Sciences, Faculty of Medicine and Health Sciences, Ramón y Cajal Institute of Sanitary Research (IRYCIS), University of Alcala, Alcala de Henares, 28871 Madrid, Spain; bjtorre@gmail.com; 2 Department of Orthopedic Surgery, University Hospital Ramón y Cajal, 28034 Madrid, Spain; 3 A + I Architecture and Engineering Ltd., 28034 Madrid, Spain; loreto@lycea.es; 4 Department of Medicine and Medical Specialities, Faculty of Medicine and Health Sciences, Ramón y Cajal Institute of Sanitary Research (IRYCIS), University of Alcala, Alcala de Henares, 28871 Madrid, Spain; basijr10@gmail.com (J.D.l.T.-M.); mjulia.bujan@uah.es (J.B.); cgbravo@amasi.es (C.G.-B.); 5 Department of Mechanical Enginneering, ICAI School of Engineering, Pontifical Comillas University, 28015 Madrid, Spain

**Keywords:** cemented–uncemented polyethylene liner, risk of wear, finite element analysis model, thickness of polyethylene, center of rotation

## Abstract

Wear debris in total hip arthroplasty is one of the main causes of loosening and failure, and the optimal acetabular fixation for primary total hip arthroplasty is still controversial because there is no significant difference between cemented and uncemented types for long-term clinical and functional outcome. To assess and predict, from a theoretical viewpoint, the risk of wear with two types of polyethylene liners, cemented and uncemented, a simulation using the finite element (FE) method was carried out. The risk of wear was analyzed according to different variables: the polyethylene acetabular component’s position with respect to the center of rotation of the hip; the thickness of the polyethylene insert; the material of the femoral head; and the relationship of the cervical–diaphyseal morphology of the proximal end of the femur to the restoration of the femoral offset. In all 72 simulations studied, a difference was observed in favour of a cemented solution with respect to the risk of wear. With regard to the other variables, the acetabular fixation, the thickness of the polyethylene, and the acetabular component positioning were statistically significant. The highest values for the risk of wear corresponded to a smaller thickness (5.3 mm), and super-lateral positioning at 25 mm reached the highest value of the von Mises stress. According to our results, for the reconstruction of the acetabular side, a cemented insert with a thickness of at least 5 mm should be used at the center of rotation.

## 1. Introduction

Total hip arthroplasty (THA) is one of the most successful and cost-effective measures in the treatment of primary arthritis of the hip [1]. On basis of data from 2000 to 2014, the frequency of THAs in the United States is projected to grow to 635,000 procedures per year by 2030 [2]. Since THAs were introduced, there has been a steady improvement in the associated technology and surgical procedure, leading to better implant survivorship [3].

The true revolution in hip surgery was introduced by Professor Sir John Charnley with his low-friction cemented polyethylene liners [4]. Initial clinical results were excellent for 10 years, but extended follow-up revealed increasing rates of failure, mostly caused by implant designs and cementing techniques [5,6]. Accordingly, cementless acetabular components were introduced with the idea of biological integration into the bone, and these have become more popular over the years. However, long-term studies with uncemented cups have proven that extensive osteolysis may also occur [7]. In addition, some hip arthroplasty registers have shown inferior survival of uncemented cups [8].

Thus, acetabular osteolysis due to polyethylene wear with subsequent aseptic loosening remains the leading cause of long-term failure in THA. Many factors affect the durability of an acetabular component. Some of these, such as surgical technique in positioning the acetabular cup, are surgeon-dependent. However, the fixation of the acetabular cup to the bone, whether it is cemented or uncemented, and the surface finish, bearing surface, head size, polyethylene thickness, and offset restoration are not [9].

To evaluate how all these factors can affect the risk of polyethylene wear, we have studied, from a theoretical point of view and using numerical analysis, the behaviour of a polyethylene liner under different variables, in an attempt to establish the true wear-risk probability and therefore predict the probability of cup failure [10,11,12,13]. This would allow us to understand the effect of implant design parameters on wear. Experimental hip simulators are not ideal for performing parametric studies, and therefore we developed a numerical wear simulation using finite element analysis (FEA). The use of FEA [14,15,16] to validate, check, or contrast experimental data [17,18] about wear rate has been used and improved from the beginning of the use of computational techniques, with reasonable accuracy of outcomes [19]. Recently, studies have combined 2D and 3D axisymmetric approaches to understand the strain energy density in metallic implants [20].

The purpose of this study was (1) to evaluate, based on FEA, the performance of the cemented and uncemented fixation of the polyethylene in order to determine the risk of wear according to different variables, both surgeon-dependent and surgeon-independent, during simulated gait and (2) to define which variables could minimize the risk of wear in THA.

## 2. Materials and Methods

An FEA model was created to obtain order-of-magnitude values of the wear tendency under conditions extrapolated to the physiological aspects of the patient, taking into account several variables.

Simulations were developed using a structural static FE analysis with isotropic behavior for the liner, using Ansys Workbench 19 R2, with the set of parameters shown in Table 1.

**Figure 1:**
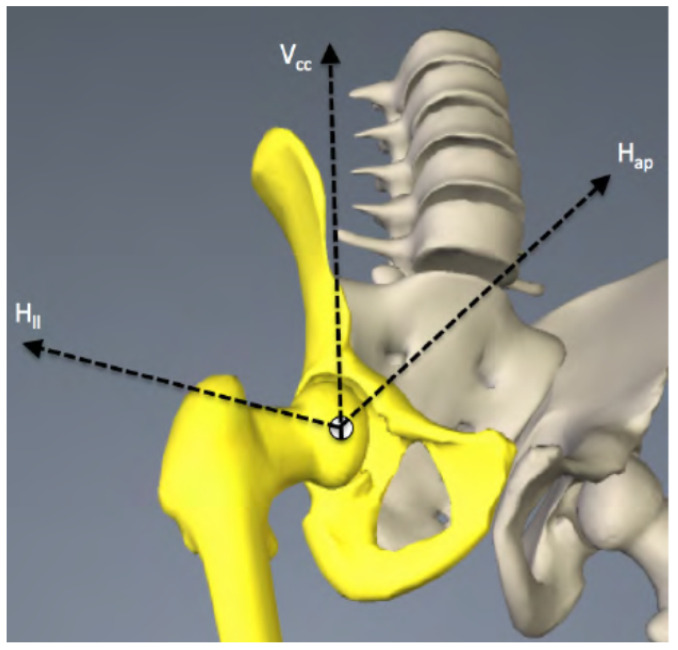
Reference axes on the hip joint. H_ll_ stands for the horizontal axis (lateral femoral), V_cc_ stands for the vertical axis (craniocaudal), H_ap_ stands for the horizontal axis (anteroposterior).

To represent the most appropriate performance according to the coxofemoral joint reality, a trihedron with three axes was defined: lateral femoral (H_II_), anteroposterior (H_ap_), and craniocaudal (V_cc_) (Figure 1).

The resultant forces (R) of contained loads were applied to the area of the polyethylene in contact with the prosthetic head. This simulated the forces acting in the hip joint for monopodial weight bearing, mimicking the physiological gait pattern. In addition, this matches Hertz’s theory regarding the femoral head–polyethylene insert friction pair. In this way, the simulations were performed using FEA, obtaining the von Mises stress distribution, which is the failure criterion used to study the wear risk.

We selected the inserts *Neutral-E1 Antioxidant Infused Polyethylene G7* acetabular system in the case of uncemented fixation and *Exceed ABT* for cemented fixation, currently on the market supplied by Zimmer Biomet in ultra-high-molecular-weight polyethylene (UHMWPE), with mechanical properties as shown in Table 2.

**Figure 2:**
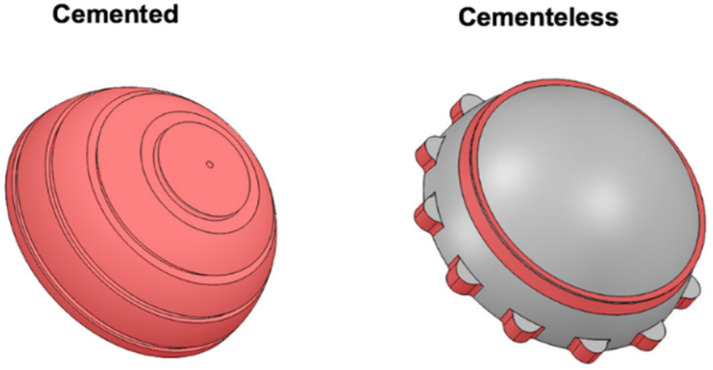
Cemented and uncemented polyethylene liner geometries. The red areas show the restriction points on the external surface for FE analysis.

Cemented and uncemented liners have their own particular geometries. The cemented insert is constrained by restrictions at the face and external edges. However, in the uncemented acetabular component, only the mechanical fixation points that the polyethylene insert has within the metal shell are restricted, to simulate the real fitting of both pieces (Figure 2).

The acetabular orientation was established with an inclination of 45° and an anteversion of 15°, as these angulations position the acetabular component in a safe way in relation to the polyethylene wear [21].

The diameter of the selected femoral head in both materials (metallic (CoCr) and ceramic (ZrO_2_)) was 32 mm. The properties of both materials are shown in Table 3. This is the most widely used diameter in clinical practice, as the use of 32 mm heads appears to offer the best compromise between joint stability and other reasons for revision [22].

We analyzed the four variables that best mimic the THA in clinical practice, to study the risk of wear through von Mises stresses. On the acetabular side: the polyethylene acetabular component´s position according to the center of rotation of the hip; the thickness of the polyethylene insert; and the femoral head material. On the femoral side: the relationship of the cervical–diaphyseal morphology of the proximal end of the femur to the restoration of the femoral offset, based on the femoral stem. All variables were investigated through a three-dimensional model.

**Figure 3:**
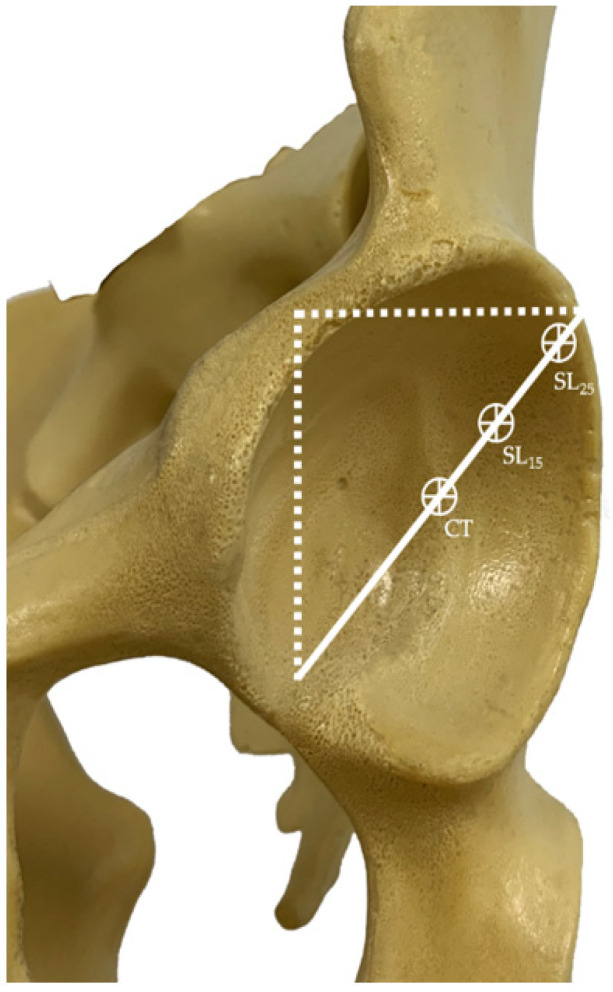
Approximate femoral head center locations and true acetabular regions according to Ranawat. CT (natural anatomical location), SL_15_ (superior and lateral location with 15 mm of displacement from CT), SL_25_ (superior and lateral location with 25 mm of displacement from CT).

To establish the wear according to the polyethylene acetabular component´s position according to the center of rotation of the hip, the real center of rotation was established according to Ranawat´s method [23]. Three references were used to position the liner. One of these was the real center of rotation (anatomical position) (CT), one was raised by 15 mm and lateralized by 5 mm with respect to the center of rotation (SL 15), and the other was raised by 25 mm and lateralized by 10 mm (SL 25) (Figure 3). Superior and lateral placement of the hip center resulted in an increase in the joint reaction forces [24]. These references represent two hip pathologies found in clinical situations: hypertrophic arthritis of the hip and high hip dysplasia.

The second variable analyzed with respect to the wear risk was the thickness of the polyethylene insert. We modeled three different geometries of polyethylene thickness (5.3 mm, 7.3 mm, and 11.3 mm). These thicknesses were determined by the manufacturer (Zimmer Biomet, Warsaw, IN, USA). A wide range was considered in order to be able to evaluate extreme values of the commercially available liners.

On the acetabular side, lastly, the wear risk according to the composition or material of the theoretical femoral head, either metal (chromium–cobalt alloy) or ceramic BIOLOX delta (zirconium oxide), was determined.

On the femoral side, the relationship of the cervical-diaphyseal morphology of the proximal end of the femur, based on the femoral stem, with volumetric wear was also studied. Two types of cervico-diaphyseal morphology of the proximal femur, represented by two stems with different neck–shaft angles were analyzed: standard stem (135°) and high-offset stem (125°). Both of these are commonly used.

In addition, the location of the wear area of both types of polyethylene liners was investigated, according to von Mises stress distributions. For this purpose, a 5.3 mm polyethylene liner thickness and a femoral ceramic head of 32 mm were chosen. This is the most frequent situation found in clinical practice.

**Figure 4:**
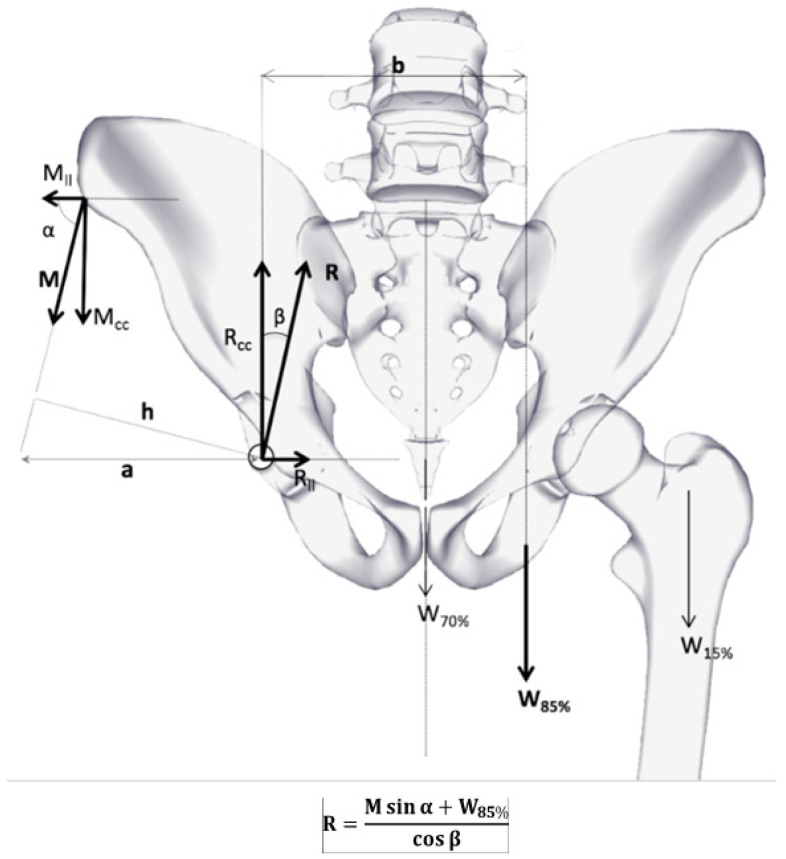
Biomechanics diagram of forces over hip (anteroposterior view). Main vectors are M (medial gluteus muscle) and R (total force over the hip joint).

To calculate the resultant forces, the static equilibrium of the coxofemoral joint was considered, together with the vectors that are associated with them. First, the vector of the pelvitrochanteric muscle group (M), which is defined by the distance from the center of the hip joint to the greater trochanter. Second, the vector that represents the body weight, (estimated at 80 kg) in monopedalism (W_85%_), which balances forces and generates a resultant force (R) on the coxofemoral joint that is finally transformed into a force per unit area (stress) on the internal face of the polyethylene (Figure 4).

In this way, as Pauwles [25] has already demonstrated by performing a free-body equilibrium, a system of first-order equations is obtained in which the resultant force´s value R is given in the equation from the picture.

To conduct the statistical analysis, a multiple regression test was carried out with all the variables already described, utilizing SPSS v 26.0 software (SPSS Inc, Chicago, IL, USA). Keeping in mind that the risk of wear was studied through the von Mises stress values, the resulting values of the stress were set as the dependent variable. In addition, an ANOVA analysis was performed on the results for the set of variables to obtain the *p*-value of each variable and therefore to determine the statistical significance of its relationship with the risk of wear. For this statistical significance, a value of 1% for the *p*-value was taken as the threshold from which the relationships between independent variables and the risk of wear were established.

## 3. Results

After the analysis carried out through a three-dimensional model and a FEA of all simulations with respect to the four types of variables studied, we obtained 72 simulations.

**Figure 5:**
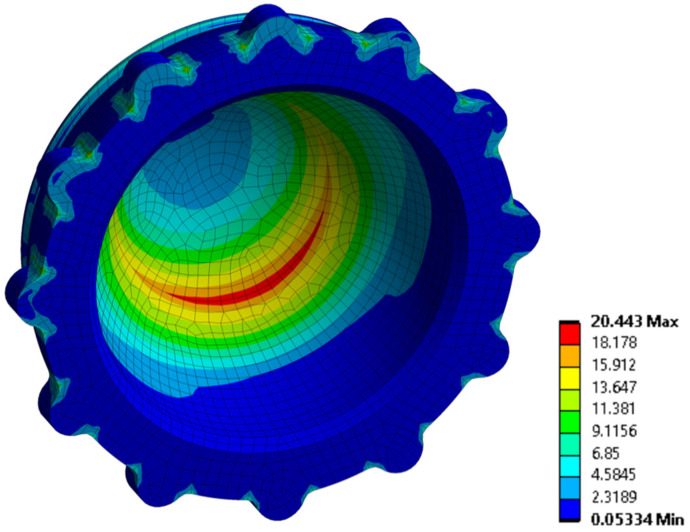
Von Mises stress distribution as a wear risk (MPa) criterion for the polyethylene liner in uncemented fixation.

The first variable analyzed was the location of the wear area on the polyethylene liner. Here, the maximum stress zones were different between the two types of inserts. In cemented implants, they were found on the internal surface of the insert, while in uncemented implants, they were along the entire rim of the insert (Figure 5).

The overall risk of wear of a cemented polyethylene liner is lower, with statistical significance.

**Figure 6:**
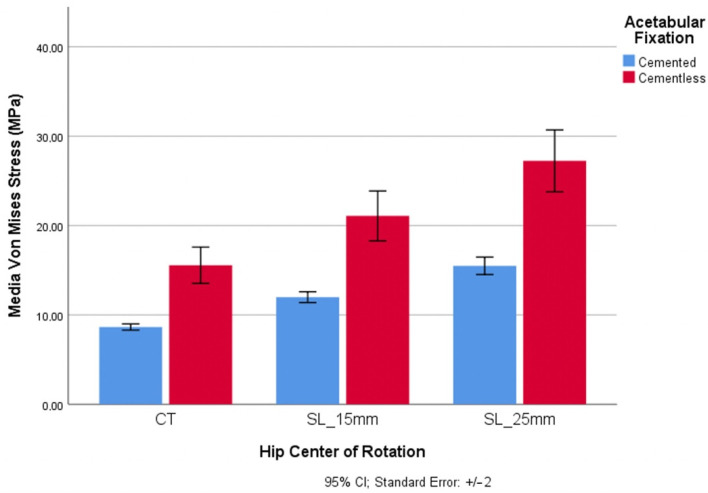
Wear risk through von Mises stress and hip center of rotation positioning: CT = anatomical position; SL_15 = superior lateralization with 15 mm vertical and horizontal distance from CT; SL_25 = superior lateralization with 25 mm vertical and horizontal distance from CT.

Regarding the polyethylene acetabular component´s position according to the center of rotation of the hip, the uncemented fixation values were higher than the cemented fixation values (Figure 6).

For the SL positioning, the mean value of the von Mises stress for the uncemented fixation reached the highest value (27.23 MPa) in SL_25mm. Regarding anatomical reconstruction (CT), the cemented and uncemented values represented were the lowest. The wear-risk values for cemented fixation were in every case lower than the uncemented values, in ranges above 55%.

Based on the effect that the von Mises stress has on polyethylene wear, the least probability of wear was seen in the anatomical position. In contrast, a higher risk of wear corresponded to an elevated and lateralized position with an uncemented fixation.

**Figure 7:**
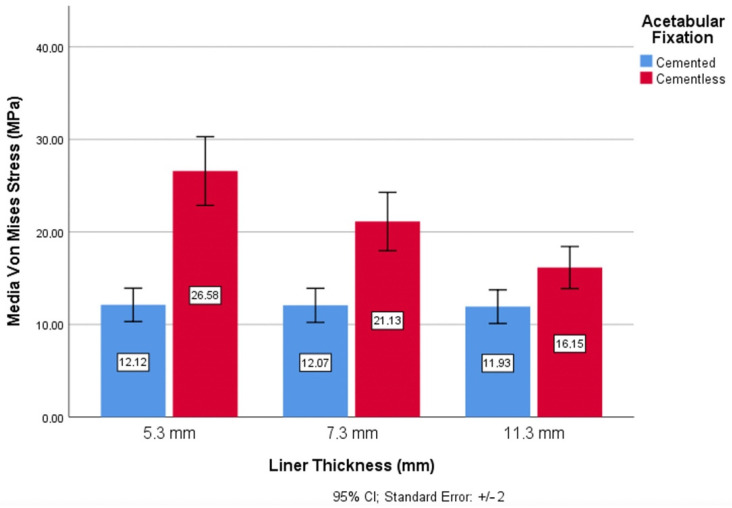
Wear risk through von Mises stress and liner thickness.

When analyzing risk of wear regarding the thickness of the polyethylene insert, relevant results were obtained. The thickness values in the cemented fixation showed little variation (0.995% between 5.3 mm and 7.3 mm thicknesses; 0.998% between 7.3 mm and 11.3 mm thicknesses; and 0.984% between 7.3 mm and 11.3 mm thicknesses). However, there was significant variation between the three thicknesses in the uncemented fixation. In this case, the highest values for the risk of wear corresponded to the smaller thicknesses, decreasing with greater thicknesses. For thicknesses of 5.3 mm, the mean von Mises stress was 26.58 MPa, which was 20.5% more than for 7.3 mm (mean von Mises stress of 21.13%) (Figure 7).

The lowest value of the von Mises stress was in the uncemented fixation with the highest thickness of polyethylene.

**Figure 8:**
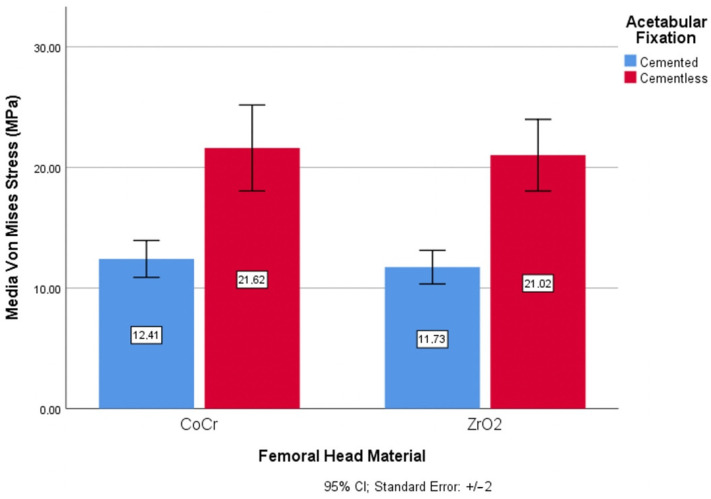
Wear risk through von Mises stress and femoral head material.

The analysis of the composition or material of the theoretical femoral head revealed a higher risk of wear in metallic materials compared to ceramics. In the comparative graphics, variations of 5.47% were seen between the materials for a cemented fixation, decreasing to 0.277% for an uncemented fixation (Figure 8).

**Figure 9:**
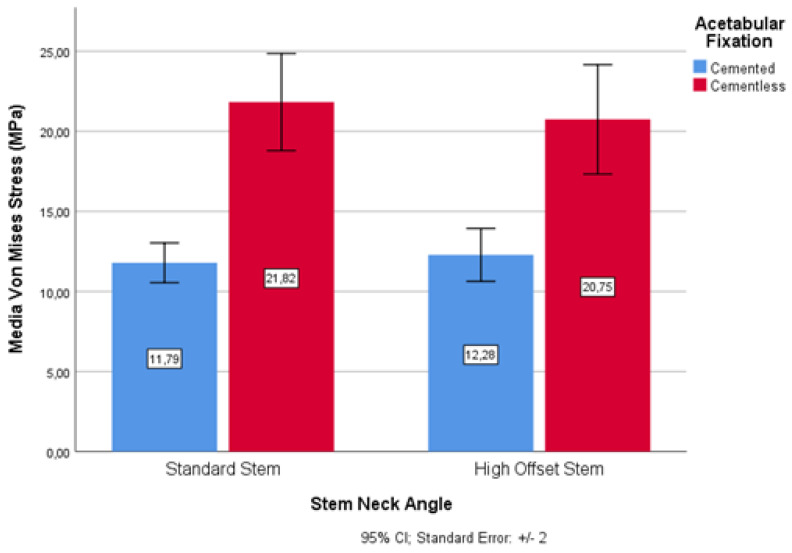
Wear risk through von Mises stress and stem neck angles.

With regard to the cervical–diaphyseal morphology of the proximal end of the femur, based on the stem neck angles, statistically significant differences between the standard stem (135°) and the high-offset stem (125°) were found in cemented and uncemented fixations (Figure 9). The standard stem had a variation of 54.03% and the high-offset stem had a variation of 59.18%.

Therefore, these two types of cervical–diaphyseal stem morphologies again showed tendencies for uncemented solutions to have a higher risk of wear than cemented solutions.

After performing the statistical analysis (ANOVA), it was shown that the acetabular fixation, thickness of the polyethylene insert, and acetabular positioning were within the range of statistical significance (*p*-value < 0.0001). There is a correlation between these variables and the risk of wear. It is worth mentioning that this is quite true regarding the fixation type (cemented–uncemented) and liner thickness. On the other hand, the head material had a *p*-value of 0.01 and, finally, the cervical–diaphyseal stem morphology exceeded *p*-value = 0.05, which implies a less statistically significant relationship (Table 4).

## 4. Discussion

In this study, we performed an FEA analysis on the risk of wear of a polyethylene liner according to different variables in THA. We developed a wear simulation method for acetabular fixation to address more complex scenarios due to both surgical and patient-based characteristics, based on FEA. FEA makes it possible to develop more comprehensive algorithms to simulate the contact behavior, gain understanding of the wear mechanics, and provide initial screening of various parameters. Few reports exist on the use of FEA to determine wear in THA [19].

Although other recent studies have developed an analysis method using an analytical model of contact pressure as a predictor of risk of wear [26], we believe that modeling many different variables using FEA provides a more reliable order-of-magnitude estimation.

Our results clearly indicate that cemented fixation of the acetabular component offers the lowest risk of wear, with a statistically significant result. This aspect is very revealing, because the current controversy regarding the optimal choice of acetabular fixation for total hip arthroplasty has its origin in the diverse outcomes reported to date [27,28]. The use of uncemented acetabular components during primary THA is on the rise in many countries. To the best of our knowledge, a study that supports the superiority in survival of uncemented acetabular components over cemented components has not previously been reported in the literature. Therefore, at present, there is a lack of sufficient evidence for superior survivorship of cementless acetabular components [29]. Current clinical studies show a wide range of heterogeneity regarding patient characteristics, materials, and bearing surfaces used, and also a wide range of study designs. The many variables introduced make it quite difficult to accurately describe the performance in isolation of acetabular components. In this scenario, the quantification that we carried out in this research could contribute to a systematic review on the evidence of wear between cemented and uncemented acetabular components [30]. From the biomechanical perspective, it must be considered that the structural behaviour of an uncemented liner has movement within the acetabular component and, therefore, greater fatigue compared to cemented inserts that act as a whole unit. This may explain the different morphological pattern of lytic lesions between cemented and uncemented inserts. In cemented components, lineal osteolysis can be observed, while a more expandable and aggressive lysis is noted for uncemented inserts [31].

We chose a fixed orientation of the liner, with an inclination of 45° and anteversion of 15°, and the diameter of the femoral head was in accordance with the real clinical situation. The diameter of the femoral head was 32 mm because this value appears to offer the best compromise between joint stability and other reasons for revision [32].

One of the major challenges in THA is the reconstruction of the acetabulum in the precise anatomic center of the hip, especially in a severely dysplasic hip. Clinically, it is well known that aseptic loosening is significantly more common and occurs by means of periacetabular osteolysis when a cup is placed superiorly to the teardrop [33]. We are not aware of any study that quantifies the risk of wear in the many different potential positions of the cup in the bony acetabulum. Although a high hip center is considered as being more than 35 mm above the intragroup [34], in the present study we selected two reference positions for the liner out of anatomical location: 15 mm and 25 mm in the superolateral position. We selected 25 mm as the maximum value because there are substantial anatomic limitations to high hip reconstruction 20 mm above the acetabular dome. Furthermore, the most unfavourable position is the placement of the insert in a raised and lateralized position. Therefore, from a clinical point of view, it is essential to reconstruct the acetabular component in its anatomical center of rotation, especially in two clinical situations: hypertrophic arthritis of the hip, where drilling must take place in a medial direction to avoid putting the center of the cup higher than the anatomical level, and high hip dysplasia.

To simulate the resultant forces in a THA, we used two types of stems with different morphologies in the neck angles. According to our results, the use of high-offset stems could reduce the wear risk of the polyethylene. This has important clinical implications. Using this type of stem could prevent the risk of dislocation in high-risk patients [35], and these stems are an essential option for restoring biomechanics and enabling a free range of motion without impinging on the activities of daily living in a substantial proportion of patients [36].

Another important clinical aspect in THA is the choice of the liner thickness. At pressent, the trend is to use large femoral heads at the expense of sacrificing the liner thickness [37]. From our study, it can be inferred that for cemented fixation, the significance of the three thicknesses is diluted, but for uncemented fixation, there is a tendency towards stress reduction in the insert as the thickness increases. Theoretically, the introduction of cross-linked PE allowed this principle to be reconsidered, since for a “hard-on-soft” bearing using cross-linked PE, the minimum thickness could be less than for a standard bearing. Unfortunately, there are no relevant data on this subject. Some authors consider that the minimum recommended thickness could be reduced to 3.9 mm with cross-linked PE. Thinner polyethylene components have demonstrated higher wear rates, although even the highest wear rate observed in the thinnest polyethylene specimen was lower than that commonly reported for non-crosslinked polyethylene components [38,39]. However, this 3.9 mm limit only partially addresses the issue of wear because it does not consider the strength, therefore it cannot yet be validated. In addition, catastrophic failures are one of the major concerns of a decrease in thickness, and the increased brittleness of highly cross-linked polyethylene puts these liners at greater risk [40]. To summarize, according to our results and taking into account the current trend towards using uncemented inserts with highly cross-linked polyethylene in clinical practice, we do not recommend the use of polyethylene with a thickness less than 5 mm.

Regarding the composition of the femoral head, there was no relevant significant difference between materials. However, we should bear in mind that the FEA does not consider the quality of the sphericity of the head. Nevertheless, the results of THAs performed for hip osteoarthritis using CoCr and ceramic femoral heads on highly cross-linked polyethylene, show no differences in the steady-state wear and no differences in clinical outcome scores [32].

Our study has limitations inherent to the FEA. First, the FEA modeling focused on wear prediction under a normal walking condition, but it was not studied during other daily activities, such as cycling, sitting down, and getting up from a chair. This is due to a lack of biomechanical information on the forces across the hip during these activities. This could be overcome by using a statistical methodology that combines a number of activities, in order to estimate the real wear during normal daily life, rather than just investigating one activity in isolation. Another limitation is that we did not consider the dynamic aspect of the acetabular orientation (e.g., pelvic tilt–lumbo-pelvic kinematics and spine-hip relationship–adjusted cup alignment). Lastly, the FEA wear analysis of the liner simulated the dry contact between bearing surfaces, not taking into account the lubrication that exists under physiological conditions. We think that the future development of fluid-structure interaction techniques could improve this aspect.

## 5. Conclusions

Although many clinical studies have been published discussing some of the aspects reviewed in the present study, to the best of our knowledge no studies have been published demonstrating wear quantification of the polyethylene liners. This is the first paper that assesses the risk of wear according the different variables during THA. The cemented fixation showed the lowest wear values, and the analysis clearly revealed that the polyethylene should be placed in an anatomical position, avoiding a high hip center. A thickness of at least 5 mm and a high-offset stem led to a decrease in the risk of wear. Based on our results, although from a purely theoretical point of view, this study could provide invaluable reference data on the effect of various parameters on the longevities of THA systems. More studies are necessary that take into account the activities of daily life.

## Figures and Tables

**Table 1:** Mesh liner parameters for FEA.

Liner Thickness (mm)	Element Type/Mesh Quality	Elements Size (mm)	Total Elements	Total Nodes	Element Accept. Ratio < 3 (%)
5.3	Solid Hexahedron/High quality	1.14319	55,010	82,156	99.1
7.3			77,960	113,960	99.1
11.3			137,728	196,708	99.5

**Table 2:** Mechanical properties for UHMWPE liner.

E ^1^ (MPa)	G ^2^ (MPa)	ν ^3^	F_y_ ^4^ (MPa)	f_u_ ^5^ (MPa) 2	Strain Max (%)
940	322	0.46	25	40	500

^1^ Modulus of elasticity; ^2^ modulus of rigidity; ^3^ Poisson’s ratio; ^4^ yield strength; ^5^ ultimate strength.

**Table 3:** Mechanical properties for the femoral head and equations of Hertz contact theory.

Head Femoral Material	E ^1^ (GPa)	ν ^2^	μ (32) ^3^
CoCr	210	0.30	0.133
ZrO_2_	358	0.24	0.096

^1^ Modulus of elasticity; ^2^ Poisson’s ratio; ^3^ friction coefficient, for 32 mm femoral head.

**Table 4:** Statistical coefficients for von Mises stress and dependent variables.

	Std. Error	*p*-Value
Acetabular Fixation	0.672	<0.0001
Cervical–Diaphyseal Morphology	0.672	0.664
Thickness Liner	0.412	<0.0001
Acetabular Component Positioning	0.273	<0.0001
Head Material	0.943	0.01

## Data Availability

The data used to support the findings of the present study are available from the corresponding author upon request.

## References

[1] Learmonth I.D., Young C., Rorabeck C. (2007). The operation of the century: Total hip replacement. Lancet.

[2] Sloan M., Premkumar A., Sheth N.P. (2018). Projected volume of primary total joint arthroplasty in the US, 2014 to 2030. JBJS.

[3] Li M., Glassman A.H. (2018). What’s new in hip replacement. JBJS.

[4] Charnley J. (1972). The long-term results of low-friction arthroplasty of the hip performed as a primary intervention. J. Bone Jt. Surg. Br. Vol..

[5] García-Cimbrelo E., Munuera L. (1992). Early and late loosening of the acetabular cup after low-friction arthroplasty. J. Bone Jt. Surg. Am. Vol..

[6] Irie T., Oyama M., Kita A., Sakamoto T., Funayama K. (2012). Medium-term result of Elite Plus hip arthroplasty: The second modular evolution of the original Charnley low-friction arthroplasty. J. Orthop. Sci..

[7] McCombe P., Williams S.A. (2004). A comparison of polyethylene wear rates between cemented and cementless cups: A prospective, randomised trial. J. Bone Jt. Surg. Br. Vol..

[8] Hailer N.P., Garellick G., Kärrholm J. (2010). Uncemented and cemented primary total hip arthroplasty in the Swedish Hip Arthroplasty Register: Evaluation of 170,413 operations. Acta Orthop..

[9] Bragdon C.R., Doerner M., Martell J., Jarrett B., Palm H., Malchau H. (2013). The 2012 John Charnley Award: Clinical multicenter studies of the wear performance of highly crosslinked remelted polyethylene in THA. Clin. Orthop. Relat. Res..

[10] Scheerlinck T. (2014). Cup positioning in total hip arthroplasty. Acta Orthop. Belg..

[11] Wang A. (2001). A unified theory of wear for ultra-high molecular weight polyethylene in multi-directional sliding. Wear.

[12] Liu F., Leslie I., Williams S., Fisher J., Jin Z. (2008). Development of computational wear simulation of metal-on-metal hip resurfacing replacements. J. Biomech..

[13] Saikko V., Calonius O. (2003). An improved method of computing the wear factor for total hip prostheses involving the variation of relative motion and contact pressure with location on the bearing surface. J. Biomech..

[14] Zietz C., Fabry C., Baum F., Bader R., Kluess D. (2015). The Divergence of Wear Propagation and Stress at Steep Acetabular Cup Positions Using Ceramic Heads and Sequentially Cross-Linked Polyethylene Liners. J. Arthroplast..

[15] Barbour P.S.M., Barton D.C., Fisher J. (1995). The influence of contact stress on the wear of UHMWPE for total replacement hip prostheses. Wear.

[16] Taylor M., Prendergast P.J. (2015). Four decades of finite element analysis of orthopaedic devices: Where are we now and what are the opportunities?. J. Biomech..

[17] Bevill S.L., Bevill G.R., Penmetsa J.R., Petrella A.J., Rullkoetter P.J. (2005). Finite element simulation of early creep and wear in total hip arthroplasty. J. Biomech..

[18] Kurtz S.M., Edidin A.A., Bartel D.L. (1997). The role of backside polishing, cup angle, and polyethylene thickness on the contact stresses in metal-backed acetabular components. J. Biomech..

[19] Wang L., Isaac G., Wilcox R., Jones A., Thompson J. (2019). Finite element analysis of polyethylene wear in total hip replacement: A literature review. Proc. Inst. Mech. Eng. Part H J. Eng. Med..

[20] Carreiras A.R., Fonseca E.M.M., Martins D., Couto R. (2020). The axisymmetric computational study of a femoral component to analysis the effect of titanium alloy and diameter variation. J. Comput. Appl. Mech..

[21] Higgins S.W., Spratley E.M., A Boe R., Hayes C.W., Jiranek W.A., Wayne J.S. (2014). A novel approach for determining three-dimensional acetabular orientation: Results from two hundred subjects. JBJS.

[22] Tsikandylakis G., Kärrholm J., Hailer N.P., Eskelinen A., Mäkelä K., Hallan G., Nord Furnes O., Pedersen A.B., Overgaard S., Mohaddes M. (2018). No increase in survival for 36-mm versus 32-mm femoral heads in metal-on-polyethylene THA: A registry study. Clin. Orthop. Relat. Res..

[23] Ranawat C.S., Dorr L.D., Inglis A.E. (1980). Total hip arthroplasty in protrusio acetabuli of rheumatoid arthritis. J. Bone Joint Surg. Am..

[24] Doehring T.C., Rubash H.E., Dore D.E. (1999). Micromotion measurements with hip center and modular neck length alterations. Clin. Orthop. Relat. Res..

[25] Pauwels F. (1976). Biomechanics of the Normal and Diseased Hip.

[26] Li G., Peng Y., Zhou C., Jin Z., Bedair H. (2020). The effect of structural parameters of total hip arthroplasty on polyethylene liner wear behavior: A theoretical model analysis. J. Orthop. Res..

[27] Maggs J.L., Smeatham A., Whitehouse S.L., Charity J., Timperley A.J., Gie G.A. (2016). The Exeter Contemporary flanged cemented acetabular component in primary total hip arthroplasty. Bone Jt. J..

[28] Gwynne-Jones D.P., Gray A.R. (2020). Cemented or uncemented acetabular fixation in combination with the Exeter Universal cemented stem: Long-term survival to 18 years. Bone Jt. J..

[29] Van Praet F., Mulier M. (2019). To cement or not to cement acetabular cups in total hip arthroplasty: A systematic review and re-evaluation. SICOT-J..

[30] Van Der Veen H.C., Van Jonbergen H.P.W., Poolman R.W., Bulstra S.K., Van Raay J.J.A.M. (2013). Is there evidence for accelerated polyethylene wear in uncemented compared to cemented acetabular components? A systematic review of the literature. Int. Orthop..

[31] Hartofilakidis G., Georgiades G., Babis G.C. (2009). A comparison of the outcome of cemented all-polyethylene and cementless metal-backed acetabular sockets in primary total hip arthroplasty. J. Arthroplast..

[32] Teeter M.G., Lanting B.A., Naudie D.D., McCalden R.W., Howard J.L., MacDonald S.J. (2018). Highly crosslinked polyethylene wear rates and acetabular component orientation: A minimum ten-year follow-up. Bone Jt. J.

[33] Georgiades G., Babis G.C., Kourlaba G., Hartofilakidis G. (2010). Effect of cementless acetabular component orientation, position, and containment in total hip arthroplasty for congenital hip disease. J. Arthroplast..

[34] Antoniades J., Pellegrini V.D. (2012). Cross-sectional anatomy of the ilium: Implications for acetabular component placement in total hip arthroplasty. Clin. Orthop. Relat. Res..

[35] Vigdorchik J.M., Sharma A.K., Elbuluk A.M., Carroll K.M., Mayman D.J., Lieberman J.R. (2021). High offset stems are protective of dislocation in high-risk total hip arthroplasty. J. Arthroplast..

[36] Weber M., Merle C., Nawabi D.H., Dendorfer S., Grifka J., Renkawitz T. (2020). Inaccurate offset restoration in total hip arthroplasty results in reduced range of motion. Sci. Rep..

[37] Muratoglu O.K., Bragdon C.R., O’Connor D., Perinchief R.S., Estok D.M., Jasty M., Harris W.H. (2001). Larger diameter femoral heads used in conjunction with a highly cross-linked ultra-high molecular weight polyethylene: A new concept. J. Arthroplast..

[38] Johnson A.J., Loving L., Herrera L., Delanois R.E., Wang A., Mont M.A. (2014). Short-term wear evaluation of thin acetabular liners on 36-mm femoral heads. Clin. Orthop. Relat. Res..

[39] Shen F.-W., Lu Z., McKellop H.A. (2011). Wear versus thickness and other features of 5-Mrad crosslinked UHMWPE acetabular liners. Clin. Orthop. Relat. Res..

[40] Tower S.S., Currier J.H., Currier B.H., Lyford K.A., Van Citters D.W., Mayor M.B. (2007). Rim cracking of the cross-linked longevity polyethylene acetabular liner after total hip arthroplasty. JBJS.

